# Supplementary material for: Content-rich biological network constructed by mining PubMed abstracts
Source: BMC Bioinformatics. 2004 Oct 8;5:147. doi: 10.1186/1471-2105-5-147 (PMC528731; doi:10.1186/1471-2105-5-147)
Supplement: Additional File 2 — The original results of the above study (non-essential files are deleted to keep the file size under the limit set by BMC bioinformatics). [file 1471-2105-5-147-S2.bz2 › chilibotAdditionalFile2/dip05/370ID9045692E6045/html/FGF7_FGFR3.html]

 


 **FGF7** and **FGFR3** 
  
Found 22 abstracts in PubMed, retrieved 05.  
 

 What does Google say? 
 PDF only 
| .edu only 

---

- Cancer Lett, 2003   **Loss of ligand binding specificity of fibroblast growth factor receptor 2 by RNA splicing in human chondrosarcoma cells.** .
  We have previously reported the alternatively spliced transcripts of fibroblast growth factor receptor FGFR 3 **[FGFR3]** derived by aberrant splicing in human cancers.
  Here, we describe a novel splice variant of FGFR2 FGFR2DeltaIII arising from skipping exons 7 10, resulting in the deletion of Ig like III domain in human chondrosarcoma cell.
  Sf9 cells expressing FGFR2DeltaIII were able to bind FGF1, FGF2, and **FGF7**, leading to loss of ligand binding specificity.
  Together with our previous findings, the present studies suggest that mRNA splicing plays an important role in the regulation of FGFRs function.

  - Cleft Palate Craniofac J, 2002   **Toward pathogenesis of Apert cleft palate FGF, FGFR, and TGF beta genes are differentially expressed in sequential stages of human palatal shelf fusion.** .
    OBJECTIVE Critical cellular events at the palatal medial edge epithelium MEE occur in unperturbed mammalian palatogenesis, the molecular control of which involves a number of growth factors including transforming growth factor beta 3 TGF beta 3 .
    Apert syndrome is a monogenic human disorder in which cleft palate has been significantly correlated to the fibroblast growth factor receptor FGFR 2 Ser252Trp mutation.
    We report the relative expression of these genes in human palatogenesis.
    METHODS The expression of the IgIIIa b and IgIIIa c transcript isoforms of FGFR2 and the proteins FGFR1, FGFR2, and **FGFR3** was studied in situ throughout the temporospatial sequence of human palatal shelf fusion and correlated with the expression of TGF beta 3.
    In addition, the immunolocalization of the ligand FGFs , and 7 was undertaken together with the intracellular transcription factor STAT1, which is activated by FGFR signaling.
    RESULTS FGFRs are differentially expressed in the mesenchyme and epithelia of fusing palatal shelves, in domains overlapping those of their ligands FGF4 and FGF2 but not **FGF7**.
    Coexpression is seen with TGF beta 3, which is implicated in MEE dynamics and FGF and FGFR upregulation, and STAT1, an intracellular transcription factor that mediates apoptosis.
    CONCLUSIONS The coregulation of molecules of the FGFR signaling pathway with TGF beta 3 throughout the stages of human palatal fusion suggests their controlling influence on apoptosis and epitheliomesenchymal transdifferentiation at the MEE.
    Experimental evidence links FGFR2 IgIIIa b loss of function with palatal clefting, and these correlated data suggest a unique pathological mechanism for Apert cleft palate.

    - Plast Reconstr Surg, 2001   **From genotype to phenotype the differential expression of FGF, FGFR, and TGFbeta genes characterizes human cranioskeletal development and reflects clinical presentation in FGFR syndromes.** .
      Mutations in the fibroblast growth factor receptor FGFR genes , and 3 are causal in a number of craniofacial dysostosis syndromes featuring craniosynostosis with basicranial and midfacial deformity.
      Great clinical variability is displayed in the pathologic phenotypes encountered.
      To investigate the influence of developmental genetics on clinical diversity in these syndromes, the expression of several genes implicated in their pathology was studied at sequential stages of normal human embryo fetal cranial base and facial ossification n = 6 .
      At 8 weeks of gestation, FGFR1, FGFR2, and **FGFR3** are equally expressed throughout the predifferentiated mesenchyme of the cranium, the endochondral skull base, and midfacial mesenchyme.
      Both clinically significant isoforms of FGFR2, IgIIIa c and IgIIIa b, are coexpressed in maxillary and basicranial ossification.
      By 10 to 13 weeks, FGFR1 and FGFR2 are broadly expressed in epithelia, osteogenic, and chondrogenic cell lineages.
      **FGFR3** is maximally expressed in dental epithelia and proliferating chondrocytes of the skull base, but poorly expressed in the osteogenic tissues of the midface.
      FGF2 and FGF4, but not **FGF7**, and TGFbeta1 and TGFbeta3 are expressed throughout both osteogenic and chondrogenic tissues in early human craniofacial skeletogenesis.
      Maximal FGFR expression in the skull base proposes a pivotal role for syndromic growth dysplasia at this site.
      Paucity of **FGFR3** expression in human midfacial development correlates with the relatively benign human mutant **FGFR3** midfacial phenotypes.
      The regulation of FGFR expression in human craniofacial skeletogenesis against background excess ligand and selected cofactors may therefore play a profound role in the pathologic craniofacial development of children bearing FGFR mutations.

      - J Neurosurg, 2001   **Negative autoregulation of fibroblast growth factor receptor 2 expression characterizing cranial development in cases of Apert P253R mutation and Pfeiffer C278F mutation syndromes and suggesting a basis for differences in their cranial phenotypes.** .
        OBJECT Heterogeneous mutations in the fibroblast growth factor receptor 2 gene FGFR2 cause a range of craniosynostosis syndromes.
        The specificity of the Apert syndrome affected cranial phenotype reflects its narrow mutational range 98% of cases of Apert syndrome result from an Ser252Trp or Pro253Arg mutation in the immunoglobulin like Ig IIIa extracellular subdomain of FGFR2.
        In contrast, a broad range of mutations throughout the extracellular domain of FGFR2 causes the overlapping cranial phenotypes of Pfeiffer and Crouzon syndromes and related craniofacial dysostoses.
        METHODS In this paper the expression of FGFR1, the IgIIIa c and IgIIIa b isoforms of FGFR2, and **FGFR3** is investigated in Apert syndrome P253R mutation and Pfeiffer syndrome C278F mutation affected fetal cranial tissue and is contrasted with healthy human control tissues.
        Both FGFR1 and **FGFR3** are normally expressed in the differentiated osteoblasts of the periosteum and osteoid, in domains overlapped by that of FGFR2, which widely include preosseous cranial mesenchyme.
        Expression of FGFR2 is restricted to domains of advanced osseous differentiation in both Apert syndrome and Pfeiffer syndrome affected cranial skeletogenesis in the presence of fibroblast growth factor FGF 2, but not in the presence of FGF4 or **FGF7**.
        Whereas expression of the FGFR2 IgIIIa b KGFR isoform is restricted in normal human cranial osteogenesis, there is preliminary evidence that KGFR is ectopically expressed in Pfeiffer syndrome affected cranial osteogenesis.
        CONCLUSIONS Contraction of the FGFR2 IgIIIa c BEK expression domain in cases of Apert syndrome and Pfeiffer syndrome affected fetal cranial ossification suggests that the mutant activation of this receptor, by ligand dependent or ligand independent means, results in negative autoregulation.
        This phenomenon, resulting from different mechanisms in the two syndromes, offers a model by which to explain differences in their cranial phenotypes.

        - Kidney Int, 2001   BACKGROUND Fibroblast growth factors are a family of at least 21 heparin binding proteins involved in many biological processes, both during development and in the adult, including cell proliferation, differentiation, and angiogenesis.
          FGFs mediate their effects through high affinity tyrosine kinase receptors , which are encoded by four genes.
          The aims of the present study were to localize FGFR 1 through FGFR 3 **[FGFR3]** in the normal adult rat kidney and to determine which functional FGFR variants and FGFs were expressed.
          METHODS Avidin biotin enhanced horseradish peroxidase immunohistochemistry was used on paraffin sections of rat kidney to localize FGFR 1 through FGFR 3 **[FGFR3]**.
          . . . reverse transcriptase polymerase chain reaction was used to examine expression of the receptor variants and also of FGF 1 through FGF 10 in cortex, outer medulla, and inner medulla.
          RESULTS By immunohistochemistry, each receptor was localized to distinct and overlapping nephron segments, such that one or more FGFRs were localized to all nephron and collecting duct epithelia.
          FGFR 1 and FGFR 3 **[FGFR3]** were localized to glomeruli, FGFR 3 **[FGFR3]** to proximal tubules and FGFR 1 to thin limbs.
          FGFR 1 through FGFR 3 **[FGFR3]** were localized to distal straight tubules, with FGFR 1 and FGFR 3 **[FGFR3]** localized to distal convoluted tubules.
          FGFR 1 and FGFR 3 **[FGFR3]** were localized to medullary collecting ducts.
          In addition, FGFR 1 was localized to the smooth muscle of renal arteries.
          All seven FGFR variants were expressed in the cortex and outer medulla, with fewer FGFRs in the inner medulla.
          FGF 1, FGF 2, FGF 7 **[FGF7]**, FGF 8, and FGF 9 were expressed in the kidney, with FGF 10 expression found only in the cortex.
          CONCLUSIONS Mapping of these receptors is critical to the determination of the effects of FGF ligands in discrete regions of the kidney.
          The distributions of the FGFRs in the normal adult kidney and the restricted expression of FGF ligands suggest that specific FGFs have distinct and important roles in the maintenance of normal kidney structure and function.
